# Supplementary figures and images for: Cloning and evaluation of reference genes for quantitative real-time PCR analysis in Amorphophallus
Source: PeerJ. 2017 Apr 26;5:e3260. doi: 10.7717/peerj.3260 (PMC5408727; doi:10.7717/peerj.3260)

**A**

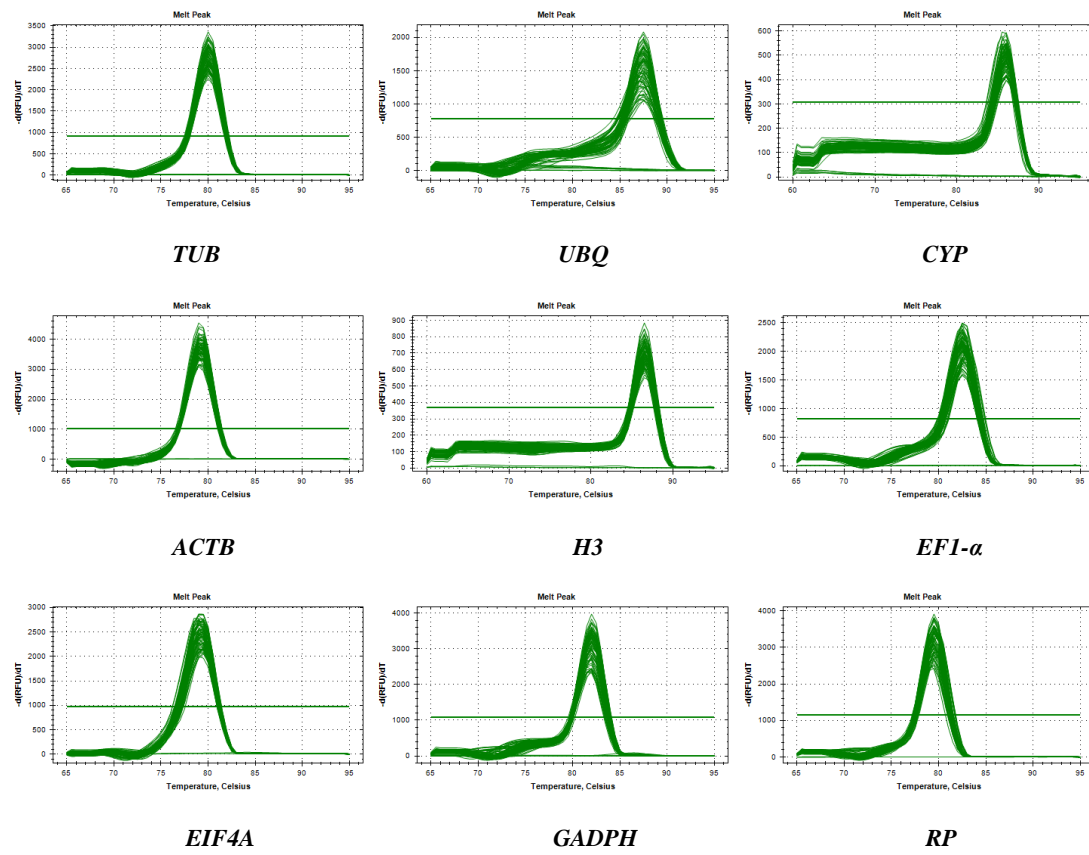

**B**

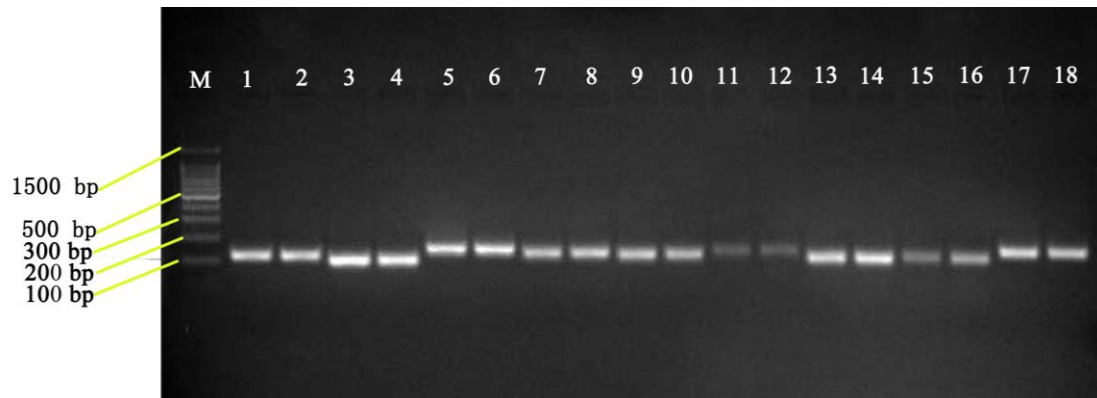

Supplement: Figure S1 — The primers were designed based on the conserved regions of related sequences in two species of Amorphophallus. (A) Melt curves of 9 candidate reference genes showing single peaks. (B) M represents 100 bp DNA ladder (Vazyme, Nanjing, China). A single band for each candidate gene in each species is shown in this picture. The candidate reference genes from left to right are: ACTB, EF1-α, UBQ, H3, TUB, CYP, RP, EIF4A, and GADPH. These genes in A. albus and in A. konjac are shown in odd lanes and even lanes, respectively. [file peerj-05-3260-s001.pdf]
