# Supplementary material for: Cloning and evaluation of reference genes for quantitative real-time PCR analysis in Amorphophallus
Source: PeerJ. 2017 Apr 26;5:e3260. doi: 10.7717/peerj.3260 (PMC5408727; doi:10.7717/peerj.3260)
Supplement: Table S1 [file peerj-05-3260-s002.docx]

**Table S1** The Cq values of 10 candidate reference genes.

|  | *ACTB* | *CYP* | *EF1-a* | *EIF4-a* | *GAPDH* | *UBQ* | *RP* | *TUB* | *H3* |
| --- | --- | --- | --- | --- | --- | --- | --- | --- | --- |
| Heat of *A.albus* | 21.37 | 25.20 | 23.62 | 25.07 | 24.32 | 23.98 | 24.45 | 26.30 | 23.90 |
|  | 20.77 | 25.72 | 23.15 | 24.28 | 23.44 | 23.41 | 23.99 | 25.72 | 24.69 |
|  | 21.62 | 28.88 | 23.55 | 25.01 | 26.36 | 24.10 | 24.48 | 33.34 | 23.99 |
|  | 20.84 | 26.47 | 23.42 | 24.57 | 24.43 | 24.09 | 24.45 | 28.66 | 26.39 |
|  | 20.17 | 23.95 | 22.52 | 23.91 | 22.47 | 23.38 | 23.62 | 25.27 | 23.61 |
|  | 22.07 | 25.10 | 23.09 | 24.55 | 23.83 | 23.36 | 22.09 | 27.60 | 23.17 |
|  | 20.78 | 24.06 | 23.84 | 24.72 | 23.11 | 22.73 | 26.04 | 26.77 | 23.74 |
|  | 20.49 | 25.88 | 23.91 | 25.36 | 25.51 | 24.97 | 22.02 | 29.41 | 24.21 |
|  | 23.05 | 24.40 | 22.66 | 23.77 | 24.15 | 23.18 | 23.28 | 27.28 | 24.83 |
|  | 20.41 | 23.79 | 21.88 | 23.55 | 22.86 | 21.66 | 19.71 | 25.86 | 23.15 |
|  | 20.82 | 24.86 | 23.97 | 25.19 | 23.51 | 24.23 | 24.46 | 28.59 | 23.65 |
|  | 22.65 | 27.63 | 24.67 | 25.99 | 25.27 | 25.55 | 24.09 | 30.65 | 25.35 |
|  | 22.48 | 26.69 | 24.68 | 24.19 | 24.56 | 25.48 | 23.04 | 28.39 | 25.48 |
|  | 20.01 | 24.65 | 23.07 | 24.16 | 24.61 | 23.64 | 23.80 | 27.40 | 23.49 |
|  | 19.97 | 23.34 | 22.92 | 23.83 | 22.84 | 23.07 | 21.14 | 26.16 | 22.67 |
| Heat of *A.konjac* | 20.04 | 24.07 | 22.99 | 23.68 | 22.62 | 22.56 | 22.16 | 26.90 | 23.10 |
|  | 21.16 | 25.00 | 23.77 | 24.58 | 25.81 | 24.27 | 19.78 | 28.14 | 24.02 |
|  | 20.48 | 24.63 | 22.71 | 23.43 | 22.30 | 21.49 | 20.43 | 25.50 | 22.32 |
|  | 20.03 | 22.50 | 23.65 | 24.23 | 24.23 | 22.46 | 20.86 | 27.38 | 22.77 |
|  | 19.54 | 22.34 | 21.91 | 23.24 | 22.22 | 21.26 | 18.97 | 25.30 | 22.24 |
|  | 22.51 | 26.24 | 24.83 | 26.01 | 23.35 | 25.09 | 22.92 | 29.64 | 25.04 |
|  | 22.26 | 24.36 | 24.12 | 24.63 | 24.52 | 23.41 | 21.79 | 28.00 | 24.32 |
|  | 21.24 | 27.03 | 24.22 | 25.08 | 23.30 | 22.96 | 22.43 | 27.23 | 23.85 |
|  | 22.08 | 24.50 | 24.68 | 24.89 | 25.60 | 24.35 | 19.62 | 28.12 | 23.99 |
|  | 20.25 | 23.49 | 23.10 | 23.09 | 22.32 | 22.56 | 18.73 | 24.99 | 22.25 |
|  | 21.27 | 25.11 | 24.78 | 24.19 | 24.66 | 23.69 | 22.19 | 28.70 | 24.26 |
|  | 20.94 | 24.90 | 24.62 | 24.28 | 24.65 | 24.14 | 23.77 | 27.40 | 23.31 |
|  | 21.68 | 24.71 | 23.65 | 24.08 | 25.23 | 23.21 | 22.14 | 27.35 | 23.58 |
|  | 21.56 | 25.24 | 24.03 | 24.07 | 25.54 | 23.32 | 21.04 | 29.31 | 23.69 |
|  | 19.70 | 23.56 | 23.28 | 23.86 | 23.18 | 22.43 | 20.84 | 27.03 | 23.17 |
| Waterlogging of  *A. albus* | 21.37 | 25.20 | 23.62 | 25.07 | 24.32 | 23.98 | 24.45 | 26.30 | 23.90 |
|  | 20.77 | 25.72 | 23.15 | 24.28 | 23.44 | 23.41 | 23.99 | 25.72 | 24.69 |
|  | 21.62 | 28.88 | 23.55 | 25.01 | 26.36 | 24.10 | 24.48 | 33.34 | 23.99 |
|  | 20.84 | 26.47 | 23.42 | 24.57 | 24.43 | 24.09 | 24.45 | 28.66 | 26.39 |
|  | 20.17 | 23.95 | 22.52 | 23.91 | 22.47 | 23.38 | 23.62 | 25.27 | 23.61 |
|  | 22.07 | 25.10 | 23.09 | 24.55 | 23.83 | 23.36 | 22.09 | 27.60 | 23.17 |
|  | 20.78 | 24.06 | 23.84 | 24.72 | 23.11 | 22.73 | 26.04 | 26.77 | 23.74 |
|  | 20.49 | 25.88 | 23.91 | 25.36 | 25.51 | 24.97 | 22.02 | 29.41 | 24.21 |
|  | 23.05 | 24.40 | 22.66 | 23.77 | 24.15 | 23.18 | 23.28 | 27.28 | 24.83 |
|  | 20.41 | 23.79 | 21.88 | 23.55 | 22.86 | 21.66 | 19.71 | 25.86 | 23.15 |
|  | 20.82 | 24.86 | 23.97 | 25.19 | 23.51 | 24.23 | 24.46 | 28.59 | 23.65 |
|  | 22.65 | 27.63 | 24.67 | 25.99 | 25.27 | 25.55 | 24.09 | 30.65 | 25.35 |
|  | *ACTB* | *CYP* | *EF1-a* | *EIF4-a* | *GAPDH* | *UBQ* | *RP* | *TUB* | *H3* |
| Waterlogging of  *A. albus* | 22.48 | 26.69 | 24.68 | 24.19 | 24.56 | 25.48 | 23.04 | 28.39 | 25.48 |
|  | 20.01 | 24.65 | 23.07 | 24.16 | 24.61 | 23.64 | 23.80 | 27.40 | 23.49 |
|  | 19.97 | 23.34 | 22.92 | 23.83 | 22.84 | 23.07 | 21.14 | 26.16 | 22.67 |
| Waterlogging of  *A. konjac* | 28.11 | 28.94 | 25.69 | 25.85 | 28.08 | 25.40 | 22.79 | 31.99 | 25.35 |
|  | 27.39 | 27.98 | 26.54 | 25.20 | 25.43 | 24.53 | 22.55 | 29.56 | 25.55 |
|  | 25.09 | 29.13 | 25.41 | 25.22 | 28.45 | 26.82 | 24.34 | 28.84 | 24.28 |
|  | 26.61 | 30.27 | 25.28 | 24.78 | 26.49 | 25.61 | 21.94 | 31.31 | 25.10 |
|  | 24.81 | 25.93 | 24.42 | 24.75 | 25.12 | 23.65 | 21.66 | 28.05 | 24.09 |
|  | 30.36 | 29.26 | 32.30 | 32.82 | 33.19 | 29.87 | 31.56 | 32.48 | 30.23 |
|  | 31.18 | 32.51 | 31.06 | 32.23 | 31.27 | 28.24 | 28.50 | 32.31 | 29.32 |
|  | 33.01 | 33.67 | 32.91 | 32.78 | 33.11 | 28.60 | 27.42 | 31.22 | 28.48 |
|  | 33.37 | 34.45 | 32.56 | 31.19 | 34.79 | 31.49 | 27.81 | 31.11 | 28.84 |
|  | 34.78 | 34.70 | 30.15 | 30.35 | 31.13 | 28.11 | 27.02 | 30.39 | 28.21 |
|  | 30.36 | 31.17 | 30.73 | 30.75 | 31.30 | 29.10 | 20.72 | 31.10 | 29.08 |
|  | 28.66 | 32.63 | 31.21 | 30.06 | 27.69 | 29.17 | 23.10 | 31.26 | 29.62 |
|  | 28.01 | 33.12 | 32.59 | 31.11 | 29.40 | 30.18 | 21.82 | 32.11 | 33.81 |
|  | 31.40 | 33.19 | 30.86 | 31.11 | 30.47 | 28.45 | 21.89 | 30.34 | 29.36 |
|  | 27.11 | 33.66 | 29.68 | 29.71 | 25.99 | 28.19 | 20.18 | 29.57 | 27.92 |
| Tissues of *A. albus* | 23.13 | 28.14 | 23.69 | 23.60 | 27.82 | 23.61 | 25.16 | 28.48 | 23.13 |
|  | 24.30 | 29.26 | 25.51 | 24.86 | 23.87 | 24.22 | 23.16 | 27.35 | 24.30 |
|  | 20.49 | 28.08 | 23.69 | 23.34 | 22.42 | 22.48 | 22.90 | 24.54 | 20.49 |
|  | 25.41 | 28.46 | 25.42 | 25.60 | 24.91 | 23.24 | 25.60 | 29.45 | 25.41 |
|  | 24.01 | 27.36 | 25.40 | 25.43 | 31.68 | 24.22 | 27.85 | 29.57 | 24.01 |
|  | 21.22 | 26.31 | 25.15 | 22.57 | 24.27 | 21.85 | 25.27 | 26.61 | 21.22 |
|  | 26.67 | 28.73 | 26.43 | 23.15 | 33.42 | 26.82 | 23.15 | 27.65 | 26.67 |
|  | 21.94 | 27.37 | 25.66 | 25.15 | 34.37 | 26.08 | 25.15 | 32.47 | 21.94 |
|  | 21.75 | 26.81 | 24.61 | 21.48 | 25.24 | 23.53 | 21.48 | 26.70 | 21.75 |
| Tissues of *A. konjac* | 25.79 | 27.63 | 25.92 | 25.52 | 26.08 | 24.28 | 27.55 | 32.09 | 27.28 |
|  | 22.64 | 27.32 | 25.48 | 25.13 | 29.62 | 27.08 | 26.55 | 28.88 | 26.31 |
|  | 19.45 | 26.50 | 25.14 | 24.12 | 25.59 | 20.93 | 23.66 | 26.34 | 25.61 |
|  | 24.88 | 27.63 | 25.14 | 25.10 | 25.20 | 25.24 | 26.55 | 31.11 | 25.74 |
|  | 22.49 | 27.76 | 23.78 | 23.35 | 26.51 | 23.34 | 27.29 | 27.48 | 26.53 |
|  | 20.17 | 26.30 | 23.64 | 23.05 | 25.04 | 21.99 | 23.86 | 25.33 | 25.26 |
|  | 26.15 | 27.09 | 24.45 | 23.59 | 31.44 | 28.22 | 25.80 | 29.62 | 26.53 |
|  | 25.44 | 27.43 | 23.60 | 22.52 | 26.49 | 24.78 | 28.98 | 28.13 | 29.12 |
|  | 22.73 | 26.73 | 23.26 | 22.33 | 25.16 | 24.13 | 24.52 | 27.46 | 23.81 |
