# Supplementary material for: Cloning and evaluation of reference genes for quantitative real-time PCR analysis in Amorphophallus
Source: PeerJ. 2017 Apr 26;5:e3260. doi: 10.7717/peerj.3260 (PMC5408727; doi:10.7717/peerj.3260)
Supplement: Sequence S1 [file peerj-05-3260-s003.docx]

>*ACTB* of *A. albus*

ATGACATGGAGAAGATTTGGCATCATACTTTCTACAACGAACTTCGAGTTGCCCCAGAGGAACACCCTGTTCTCTTAACTGAAGCCCCCCTTAACCCAAAGGCCAACAGAGAGAAGATGACACAGATCATGTTTGAAACATTCAATGTTCCAGCAATGTATGTTGCAATTCAGGCCGTCCTCTCTTTATATGCTAGTGGCCGTACGACAGGTATTGTGCTGGATTCTGGTG

>*ACTB* of *A. konjac*

ACGACATGGAGAAAATTTGGCATCATACTTTCTACAACGAACTTCGAGTTGCCCCAGAGGAACACCCTGTTCTCTTAACTGAAGCCCCCCTTAACCCAAAGGCCAACAGAGAGAAGATGACACAGATCATGTTTGAAACATTCAATGTTCCAGCAATGTATGTTGCAATTCAGGCCGTCCTCTCTTTATATGCTAGTGGCCGTACGACAGGTATTGTGCTGGATTCTGGTG

>*H3* of *A. albus*

GGTGAAGAAGCCCCACCGGTTTAGGCCTGGGACCGTTGCACTCCGTGAGATCCGCAAGTATCAGAAGAGCACGGAGTTGCTCATCCGGAAGCTGCCATTCCAGCGCCTCGTTCGGGAGATCGCTCAGGACTTCAAGACCGACCTCCGGTTCCGGAGCTCCGCGGTGTCTGCGCTCCAGGAGGCTGCCGAGGCCTATCTGGTGGGGCTCTTCGAGGACACCAATCTCTGCGCCATCCACGCCAAGCGCGTCACCATCATGCCCAAGGACATCCAGCTTGCCCGT

>*H3* of *A. konjac*

GGTGAAGAAGCCCCACCGGTTCAGGCCTGGGACCGTTGCACTCCGTGAGATCCGCAAGTATCAGAAGAGCACGGAGTTGCTCATCCGGAAGCTGCCATTCCAGCGCCTCGTTCGGGAGATCGCTCAGGACTTCAAGACCGACCTCCGGTTCCAGAGCTCCGCGGTGTCTGCGCTCCAGGAGGCTGCCGAGGCCTATCTGGTGGGGCTCTTCGAGGACACCAATCTCTGCGCCATCCACGCCAAGCGCGTCACCATCATGCCCAAGGACATCCAGCTTGCCCGT

>*CYP* of *A. albus*

GAGAACTTCCGGGCGCTCTGCACCGGCGAGAAGGGGGTGGGCCGCTCCGGCAAGCCCCTCCACTACAAGGGCTCCACCTTCCACCGGGTGATCCCCCAGTTCATGTGTCAGGGCGGCGACTTCACCGCCGGGGACGGCACCGGCGGCGAGTCCATCTACGGCGCCAAGTTCGAGGACGAGAACTTCGTGAAGAAGCACACCGGCCCCGGGGTGCTGTCCATGGCCAACGCTGGCCCTGGGACCAACGGGTCCCAGTTCTTCATCTGCACGGAGAAGACGTCGTGGCTTGACGGGAAGCACGTCGTGTTCGGCCAGGT

>*CYP* of *A. konjac*

GAGAACTTCCGGGCGCTCTGCACCGGCGAGAAGGGGGTGGGCCGCTCCGGCAAGCCCCTCCACTACAAGGGCTCCACCTTCCACCGGGTGATCCCCCAGTTCATGTGTCAGGGCGGCGACTTCACCGCCGGGAACGGCACCGGCGGCGAGTCCATCTACGGCGCCAAGTTCGAGGACGAGAACTTCGTGAAGAAGCACACCGGCCCCGGGGTGCTGTCCATGGCCAACGCTGGCCCTGGGACCAACGGGTCCCAGTTCTTCATCTGCACGGAGAAGACGTCGTGGCTTGACGGGAAGCACGTCGTGTTCGGCCAGAT

>*GADPH* of *A. albus*

CAAAGGCAAGGCTGCTGCTCATTTAAAGGGTGGTGCCAAGAAGGTCGTCATCTCAGCTCCTAGCAAAGATGCTCCCATGTTTGTTGTTGGTGTAAATGAGCATGAGTACAAGCCAGATATTGATATTGTATCTAATGCTAGCTGTACCACTAACTGCCTCGCTCCTCTGGCCAAGGTTATTAATGACAGATTTGGTATTGTTGAGGGTTTGATGACCACTGTGCACTCTATCACTGCTACCCAGAAGACTGTCGATGGACCTTCTGCCAAGGACTGGAGGGGTGGAAGGGCTGCATCATTCAACATCATTCCCAGCAGCACTGGTGCCGCCAAG

>*GADPH* of *A. konjac*

CAAAGGCAAGGCTGCTGCTCATTTAAAGGGTGGTGCCAAGAAGGTCGTCATCTCAGCTCCTAGCAAAGATGCTCCCATGTTTGTTGTTGGTGTAAATGAGCATGAGTACAAGCCAGATATTGATGTTGTATCTAATGCTAGCTGTACCACTAACTGCCTCGCTCCTCTGGCCAAGGTTATTAATGACAGATTTGGTATTGTTGAGGGTTTGATGACCACTGTGCACTCTATCACTGCTACCCAGAAGACTGTCGATGGACCTTCTGCCAAGGACTGGAGGGGTGGAAGGGCTGCATCATTCAACATCATTCCCAGCAGCACTGGTGCCGCCAAG

>*EF1-α* of *A. albus*

GACTGCCACACCTGACACATTGCCGTCAAGTTTGCGGAGATCCTGACAAAGATCGATAGGCGTTCTGGTAAGGAGCTGGAGAAGGAGCCCAAGTTCCTGAAGAATGGCGATGCTGGGTTTGTGAAGATGATCCCCACCAAGCCCATGGTGGTGGAGACTTTCTCCGAGTACCCCCCACTTGGTAGGTTTGCCGTGAGGGACATGAGGCAGACGGTCGCGGTCGGTGTCATCAAGAGCGTCG

>*EF1-α* of *A. konjac*

GACTGCCACACCTCACACATTGCCGTCAAGTTTGCGGAGATCCTGATAAAGATCGATAGGCGTTCTGGTAAGGAGCTGGAGAAGGAGCCCAAGTTCCTGAAGAATGGCGATGCTGGGTTTGTGAAGATGATCCCCACCAAGCCCATGGTGGTGGAGACTTTCTCCGAGTACCCCCCACTTGGTAGGTTTGCCGTGAGGGACATGAGGCAGACGGTCGCGGTCGGTGTCATCAAGAGTGTCG

>*EIF4A* of *A. albus*

GATGAGCTGACCCTTGAGGGTATCAAGCAGTTCCATGTGAATGTTGACAAGGAGGACTGGAAACTTGAGACCCTCTGCGATCTCTACGAAACCTTGGCCATCACCCAGAGCGTCATCTTTGTCAACACCCGGCGCAAGGTTGACTGGCTTACTGACAAGATGAGGAGCAGGGATCACACAGTCTCAGCTACTCATGGAGATATGGACCAGAACACTAGGGACATCATTATGAGGGAGTTTAGGTCAGGGTCTTCTCGTGTCCTTATCACCACTGATCTTCTCGCTCGTGGTATTGATGTCCAGC

>*EIF4A* of *A. konjac*

GATGAGCTGACCCTTGAGGGTATCAAGCAGTTCTATGTGAATGTTGACAAGGAGGACTGGAAACTTGAGACCCTCTGCGATCTCTACGAAACCTTGGCCATCACCCAGAGCGTCATCTTTGTCAACACCCGGCGCAAGGTTGACTGGCTTACTGACAAGATGAGGAGCAGGGATCACACAGTCTCAGCTACTCATGGAGATATGGACCAGAACACTAGGGACATCATTATGAGGGAGTTTAGGTCCGGGTCTTCTCGTGTCCTTATCACCACTGATCTTCTCGCTCGTGGTATTGATGTTCAGC

>*TUB* of *A. albus*

CGCCCTGACAACTTCGTCTTCGGGCAGTCTGGCGCCGGCAACAACTGGGCCAAGGGGCACTACACCGAGGGCGCCGAGCTGATCGACTCCGTCCTCGACGTCGTCCGCAAGGAGGCCGAGAATTGCGATTGCCTGCAAGGATTCCAGGTATGCCATTCATTGGGAGGTGGCACTGGATCAGGCATGGGAACATTGCTCATTTCCAAGATCAGGGAGGAGTATCCCGACCGCATGATGCTGACCTTCTCTGTCTTCCCATCACCAAAGGTGTCTGATACCGTTGTGGAACCATACAATGCTACACTTTCAGTCCACCAGCTGGTTGAGAATGCCGATGAATGCATGGTACTTGACAATGAAGCTCTATATGATATTTGCTTCCGCACTCTAAAGCTTGCTACACCTACCTTTGGCGATCTCAATCACCTTATTTCTGCCACAATGAGTGGAATTACTTGCTGCCTCCGTTTCCCCGGCCAGCTCAACTCCGACCTCAGGAAGCTGGCTGTGAATCTAATTCCATTCCCCCGTCTCCACTTCTTCATGGTTGGATTTGCGCCGCTCACCTCTAGGGGCTCCCAACAGTACCGTGCTCTCACTGTGCCCGAGCTGACCCAGCAGATGTGGGACTCCAAG

>*TUB* of *A. konjac*

CGCCCCGACAACTTCGTCTTCGGGCAGTCTGGCGCCGGCAACAACTGGGCCAAGGGGCACTACGCCGAGGGCGCCGAGCTGATCGACTCCGTCCTCGACGTCGTCCGCAAGGAGGCCGAGAATTGCGATTGCCTGCAAGGATTCCAGGTATGCCATTCATTGGGAGGTGGCACTGGATCAGGCATGGGAACATTGCTCATTTCCAAGATCAGGGAGGAGTATCCCGACCGCATGATGCTGACCTTCTCTGTCTTCCCATCACCAAAGGTGTCTGATACCGTTGTGGAACCATACAATGCTACACTTTCAGTCCACCAGCTGGTTGAGAATGCCGATGAATGCATGGTACTTGACAATGAAGCTCTATATGATATTTGCTTCCGCACTCTAAAGCTTGCTACACCTACCTTTGGTGATCTCAATCACCTTATTTCTGCCACAATGAGTGGAATTACTTGCTGCCTCCGTTTCCCCGGCCAGCTCAACTCCGACCTCAGGAAGCTGGCTGTGAATCTAATTCCATTCCCCCGTCTCCACTTCTTCATGGTTGGATTTGCGCCGCTCACCTCTAGGGGCTCCCAACAGTACCGTGCTCTCACTGTGCCCGAGCTGACCCAGCAGATGTGGGATGCCAAG

>*UBQ* of *A. albus*

TAACGGGGAAGACCATCACGCTGGAGGTGGAGTCCTCGGACACCATCGACAACGTGAAGGCCAAGATCCAGGACAAGGAGGGCATCCCACCGGACCAGCAGCGGCTCATCTTCGCCGGGAAGCAGCTCGAGGACGGCCGCACCCTTGCCGACTACAACATCCAGAAGGAGTCCACCCTCCACCTCGTCCTCCGCCTCCGCGGCGGCGCCAAGAAGCGCAAGAAGAAGACCTACACCAAGCCCAAGAAGATCAAGCACAAGAAGAAGAAGGTCAAGCTCGCTCTCCTCCAGTTTTACAAGGT

>*UBQ* of *A. konjac*

TTGCGGGGAAGACCATCACGCTGGAGGTGGAGTCTTCGGACACCATCGACAACGTGAAGGCCAAGATCCAGGACAAGGAGGGCATCCCACCGGACCAGCAGCGGCTCATCTTCGCCGGGAAGCAGCTCGAGGACGGCCGCACCCTTGCCGACTACAACATCCAGAAGGAGTCCACCCTCCACCTCGTCCTCCGCCTCCGCGGCGGCGCCAAGAAGCGCAAGAAGAAGACTTACACCAAGCCCAAGAAGATCAAGCACAAGAAGAAGAAGGTCAAGCTCGCTCTCCTCCAGTTCTACAAGGT

>gi|254998327:1-359 Amorphophallus konjac chloroplast gene for ribosomal protein L16, partial cds

TGAAAGGAATATCTTATAGAGGCAATCATATTTGTTTTGGAAGATACGCTCTTCAGGCACTTGAACCCGCTTGGATCACAGCTAGACAAATAGAAGCGGGACGAAGAGCAATGACCCGATATGCACGTCGTGGTGGAAAAATATGGGTACGTATATTTCCCGACAAACCTGTTACAGTAAGACCTACAGAAACACGTATGGGTTCGGGGAAAGGGTCTCCCGAATATTGGGTATCTGTTGTTAAACCGGGTCGAATACTTTATGAAATGGGCGGAGTATCCGAAACTGTGGCCAGAGCAGCCATTTCAATAGCTGCGTGCAAAATGCCTATACGAACTCAATTTATTATTGCGGGATAG

>Verified sequence of RP16 in *A. albus*

TCAGGCACTTGAACCCGCTTGGATCACAGCTAGACAAATAGAAGCGGGACGAAGAGCAATGACCCGATATGCACGTCGTGGTGGAAAAATATGGGTACGTATATTTCCCGACAAACCTGTTACAGTAAGACCTACAGAAACACGTATGGGTTCGGGGAAAGGGT

>*SHSP* of *A.albus*

ATGGACGTCAGGATGTTGGGACTGGAGAACCCGCTGTTCTCGGCGCTGCATCATCTGATGGACGTCCCGGAGGAGATGGGGAAGGCCTTCAACGCCCCAACCCACGCCTACGTCAGGGACGCCAAGGCCATGGCCTCCACCCCTGCCGACGTCAAGGAGTACCCCAACTCCTACGTCTTCGTCGTCGACATGCCGGGGCTCAAGTCCGGCGAGATCAAGGTCCAGGTGGAGGACGGCAACGTGCTGGTCATCAGCGGCGAGCGCAAGCGGGAGGAGGACAAGGACGGCAAGTACCTGCGCATGGAGCGCAGGGTGGGCAAGTTCATGAGGAAGTTCTCGCTGCCGGACAACGCCAACACGGACGCCGTCTCCGCCGTGTGCCAGGACGGGGTGCTCACCGTCACCGTGCAGAAGCTGCCGCCTCCCGAGCCCAAGAGGCCCAAGACCGTCGAAGTCAAGGTCGTCTAA

>*SHSP* of *A.konjac*

ATGGACGTCAGGATGTTGGGACTGGAGAACCCGCTGTTCTCGGCGCTGCATCATCTGATGGACGTCCCGGAGGAGATGGGGAAGGCCTTCAACGCCCCAACCCACGCCTACGTCAGGGACGCCAAGGCCATGGCCTCCACCCCTGCCGACGTCAAGGAGTACCCCAACTCCTACGTCTTCGTCGTCGACATGCCGGGGCTCAAGTCCGGCGAGATCAAGGTCCAGGTGGAGGACGGCAACGTGCTGGTCATCAGCGGCGAGCGCAAGCGGGAGGAGGACAAGGACGGCAAGTACCTTCGCATGGAGCGCAGGGTGGGCAAGTTCATGAGGAAGTTCTCGCTGCCGGACAACGCCAACACGGACGCCGTCTCCGCCGTGTGCCAGGACGGGGTGCTCAGCGTCACCGTGCAGAAGCTGCCGCCGCCCGAGCCCAAGAGGCCCAAGACCGTCGAAGTCAAGGTCGTCTAA
